# Alternative Splicing of Transcription Factors' Genes: Beyond the Increase of Proteome Diversity

**DOI:** 10.1155/2009/905894

**Published:** 2009-07-12

**Authors:** David Talavera, Modesto Orozco, Xavier de la Cruz

**Affiliations:** ^1^Barcelona Science Park (PCB), Molecular Modelling and Bioinformatics Unit, Institute for Research in Biomedicine (IRB), 08028 Barcelona, Spain; ^2^European Bioinformatics Institute (EMBL), Wellcome Trust Genome Campus, Hinxton, Cambridge CB10 1SD, UK; ^3^Faculty of Life Sciences, University of Manchester, Manchester M13 9PL, UK; ^4^Barcelona Science Park (PCB), Protein Structure and Modelling Node, The Spanish National Bioinformatics Institute, Genoma España, 08028 Barcelona, Spain; ^5^Departament de Bioquímica i Biologia Molecular, Facultat de Biologia, Universitat de Barcelona, 08028 Barcelona, Spain; ^6^Department of Life Sciences, Barcelona Supercomputing Center, 08034 Barcelona, Spain; ^7^Institució Catalana de Recerca i Estudis Avançats (ICREA), 08010 Barcelona, Spain; ^8^Consejo Superior de Investigaciones Cientificas, Institut de Biologia Molecular de Barcelona, 08028 Barcelona, Spain

## Abstract

Functional modification of transcription regulators may lead to developmental changes and phenotypical differences between species. In this work, we study the influence of alternative splicing on transcription factors in human and mouse. Our results show that the impact of alternative splicing on transcription factors is similar in both species, meaning that the ways to increase variability should also be similar. However, when looking at the expression patterns of transcription factors, we observe that they tend to diverge regardless of the role of alternative splicing. Finally, we hypothesise that transcription regulation of alternatively spliced transcription factors could play an important role in the phenotypical differences between species, without discarding other phenomena or functional families.

## 1. Introduction

Some years ago the hypothesis that morphological differences between species are due to changes in the gene regulatory regions was proposed [1]. Recent advances in the development field are providing supporting evidences even when comparing relatively remote species [2] and their interpretation within an evolutionary context has lead to creation of the evo-devo field. An important part of the research within this field is focused on the comparison of gene regulatory regions [2]. However, less attention has been devoted to explore the role of transcription factors (TFs). Regulation of the activity of transcription factors is a complex process [3] including a broad range of intrinsic and environmental factors. This is particularly relevant within the context of development and evolutionary research, since control of the amount of TFs, at precise locations and times, may constitute a finer alternative to the more drastic presence/absence of TFs binding sites. 

Among the mechanisms modulating the activity of TFs the role of alternative splicing (AS) has been well documented in the recent years [3–5]. Indeed, different studies have shown that AS of TFs results in regulatory isoforms [3, 6–9] that can be tissue- or development stage-specific [4, 10–13] and show cell distribution variation [14]. In general, the biological effect of AS on TFs can be easily interpreted if we consider the fact that TFs are commonly large proteins with a modular composition [4, 7]. TFs domains have different roles related to the main function of TFs: DNA binding, dimerization and function regulation. DNA binding domains are required to recognize target sequences; dimerization domains allow the building of dimers or oligomers which are the biological unit of many TFs; and regulatory domains are used to detect external stimulus or signals from transduction pathways. Therefore, loss of one of these domains will be accompanied by the loss of one of these functional properties, thus resulting [3, 15–18] in transcripts that either (i) lack the original activity, (ii) show and increase or decrease in this activity, or (iii) act as dominant negative of the fully-functional isoform, having an antagonistic effect. Obviously, the nature of the regulation associated to AS depends on the domains involved. However, it is important to notice that a partial deletion or substitution can also lead to a loss or modification of domain functionality [19, 20].

Bioinformatics research has widely studied AS using several approaches, contributing to shape our present view of the functional changes caused by this phenomenon [21–29]. In the case of TFs, work from different authors has focused in the study of AS mechanisms of cancer-related genes [30], in the properties of specific domains in AS [31], and in the role of AS of TFs in different species [10]. Among other facts, it has been established that human and mouse have different TFs variants [31] and that TFs seem to be more frequently spliced, creating tissue-specific isoforms with different domain architectures [10]. In spite of their growing amount, the view provided by these studies is still incomplete, and important aspects related to the variability generated by AS and its interspecies conservation remain unclear.

In this article we compare how AS of TFs varies between human and mouse, two species with clear morphological differences, focusing on some characteristics related to the generation of regulatory mechanisms. In addition, we also compared the expression levels of orthologous TFs to see whether there are substantial expression differences between both species. Our results indicate that human and mouse use similar mechanisms to regulate the action of TFs. We also find, for a population of human-mouse orthologs, that TFs tend to show diverging gene expression changes not related to the presence of AS.

## 2. Materials and Methods

### 2.1. Data

TFs and enzymes sequences and position of variable regions were obtained from SwissProt [32]. The most important point in the bioinformatics studies is the election of the dataset. There are several options: (1) manually curated databases; (2) automatically-annotated databases and (3) mining of publicly available experimental data, all having strengths and weaknesses [33]. Curated databases contain the most reliable information, but have a small coverage. Automatic annotation of databases increases the amount of data, but as the annotations are transferred by homology their reliability depend on the used thresholds. Finally, the public repositories contain a huge amount of unprocessed data. Obviously, all achieved conclusions should be nuanced according to the used dataset. As our analysis relied in the correlation between alternative regions and functional domains, we thought that it was crucial to analyse real isoforms, which are provenly transcribed and translated, instead of putative peptides. Consequently, we chose the database containing less artefacts [34]. In addition, although we missed several isoforms in our analyses, the general trends should not be importantly affected, as we found in a previous study [26].

### 2.2. Similarity of TFs

We compared all TFs isoforms using the CD-HIT software [35]. This tool clusters all sequences at a given identity threshold.

### 2.3. Domain Predictions

Functional domains from SMART [36] and Pfam [37] were identified with RPS-BLAST (a PSI-BLAST variant [38]) using 0.02 as *E*-value and the low complexity filter. When the domain annotation for these two databases overlapped without completely coinciding, we chose the longest domain assignment and discarded the shortest if at least 60% of it overlapped with the longest.

### 2.4. Precision of AS Effects on TFs

For each AS modification, we took the N- and C-terminal positions of the variable region and the N- and C-terminal boundaries of the related functional domains, if any. We ordered the four positions and calculated the precision as the ratio between the residues being both functional and alternatively spliced and all the residues being either in the functional domain or the AS region. A precision close to 1 meant that AS was almost coincident with functional domain boundaries, whereas a low precision showed a lower correlation.

### 2.5. TFs Expression Patterns

Expression data for human and mouse genes were retrieved from the SymAtlas server (http://symatlas.gnf.org, now at https://biogps.gnf.org/) (human: GNF1H; mouse: GNF1M) [39]. These data did not contain isoform expression, but all isoforms were grouped. This fact was useful to our goal, because we were not interested in the expression of the equivalent isoforms, but in the effect of the ability of alternatively splice genes on the expression pattern of orthologs. 

We analysed 559 TFs from 30 common tissues (mouse spinal cord data was the average of upper and lower spinal chord ones). This dataset was divided in three sets, depending on the presence of AS, as follows: (a) a set of 123 TFs for which both orthologs had AS; (b) a set of 109 TFs which were alternatively spliced in one species but not in the other; and finally (c) a set of 327 TFs with no AS neither in human nor in mouse. In addition, we analysed the expression pattern of 1923 enzymes so as to study if TFs had some kind of specific features.

Gene expression tissue patterns were compared using the Pearson's correlation, following similar studies [40–43]. High correlations mean that genes have similar expression patterns, whereas low correlations indicate variations due to tissue-specific expression. As suggested by Liao and Zhang [43], we used relative abundance instead of signal intensity measured from the microarrays. Signal intensity does not quantify the abundance of mRNA in the sample, is different for each experiment and is influenced by many factors. On the contrary, the relative abundance normalises the values within each experiment: briefly, each expression level is divided by the sum of all the signals in the experiment. This normalisation allows comparison of results of different experimental results. When several replicas were available for a given experiment we averaged the expression data. In addition, comparison between these replicas was utilized to obtain a control of the reproducibility of the results. In absence of alternative experimental validation, this control ensures that the possible differences are not due to the variance between experiments.

## 3. Results and Discussion

### 3.1. Diversity of TFs

Since the publication of the human genome it was suggested that rate differences in AS could be associated to phenotypic differences between organisms [44]. In the case of TFs, Taneri and colleagues [10] have recently shown that for mouse the percentage of genes with AS is higher for TFs than for other proteins. In our case (Table 1) we found that this was the case for mouse (*T*-test *P*-value ~0) as well as for human (*T*-test *P*-value ~0.04). This means that the higher rate of AS in TFs could be a general feature. In addition, the average number of isoforms per gene was similar in both species. The actual percentages of genes having AS were different from those obtained by Taneri and colleagues (62% and 29% of TFs loci and all loci, resp.) [10], because they build their database using a computational algorithm, whereas we relied only on experimentally validated protein isoforms. The important point is that the biases affecting the manual or automatic annotation of AS would affect all loci. Thus, independently of the presented percentages, the fact is that TFs genes have a significantly higher rate of AS. The rate differences between human and mouse could be real or simply due to the different coverage of the two species [45, 46].

Additionally, we looked at the similarity of TFs. Given the existence of structural and evolutionary restrictions [47, 48] and that the number of functional domains is limited; we expected that many TFs would share a big part of their sequence. However, we observed that very few TFs had a high identity percentage neither in human nor in mouse (Table 2). Moreover, just one third or one quarter of the isoforms was at least 40% identical, meaning that the majority of TFs probably did not have the same functionality [49]. The latter result was not different from that obtained with the control; however, control included all human non-TFs proteins, which could be very different proteins. According to the literature, no big differences would be expected if using mouse proteins instead of the human dataset as a control [26, 27, 45, 50, 51]. These results suggest that the role of AS in the slight regulation of TFs is almost unique, as gene duplicates are so divergent that they hardly could play similar roles [26].

### 3.2. AS Effects on TFs Functional Domains

Next we studied how AS affects domain composition in TFs, a feature directly related to the regulation of protein function in general [26, 27, 52–54] and to that of TFs in particular [4, 5, 16, 55]. Analysing TFs with AS from our dataset, we found that in 28% and 18% of human and mouse TFs at least one isoform showed domain composition changes; and from the different domain types present, 59% in human and 68% in mouse were affected by alternative splicing. These data suggest that both organisms use AS to regulate the action of TFs in a similar way. 

As the effect of AS upon the TFs depend on the affected functional domains, it is important to know whether the process is random-like. In our case we first observed that AS did not affect all possible domains. And second, we found that among those affected, not all were affected with the same frequency, in accordance with what was found by Liu and Altman [25] when considering the whole proteome. Moreover, we see that the most affected domains (HOX, HOLI, HLH, and PHD) are the same and with similar percentages in both species. The only exception was the (Krueppel-associated box) KRAB domain, which is frequently affected in human and not in mouse, probably due to the absence of mouse orthologs in our dataset. In accordance with Taneri and colleagues [10], we find that AS frequently affects DNA-binding domains in both species. For example, the most frequently affected domain common to both species is HOX, the homeobox domain responsible of DNA binding in transcription factors. However, we found that AS may also affect domains with other biochemical properties. This is the case of the common second most frequently affected domain, HOLI, a hormone-binding domain present in nuclear hormone receptors. Also a frequently spliced-out domain, PHD, is involved in protein-protein interactions and/or interactions with chromatin. 

**Figure 1:**
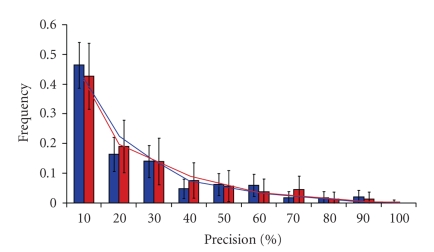
The four possible types of overlap between AS (coloured in red) and protein domains (boxes).

**Figure 2:**
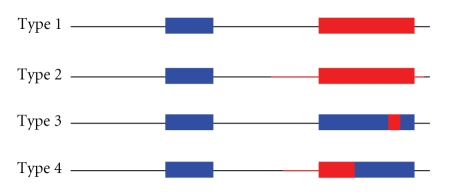
Precision of AS effect on TFs functional domains. Bar histograms show results for the observed frequencies, whereas the lines show distribution for the expected frequencies at random. Human (blue) and mouse (red) results are shown.

Previous studies on the effect of AS on functional domains indicate that AS shows a bias towards encompassing whole domains [29, 56]. However, as emphasized by Zavolan and van Nimwegen [57] there are also many cases in which AS only affects part of the protein domain. To explore what happened in the case of TFs, we measured the overlap between AS and domain boundaries. Firstly, we measured the precision of AS effect on TFs. Surprisingly; results show a random distribution either in human or mouse (Figure 1). As alternative splicing is determined by gene structure and cannot occur wherever by chance, we refined our analysis. More precisely we considered four different situations (Figure 2): (I) AS and domain boundaries coincide exactly; (II) the spliced-out region spreads over the whole domain and surrounding sequence; (III) the spliced-out region is completely included within the domain and (IV) there is only a partial overlap between the spliced-out region and the functional domain. A high frequency of type I cases would mean a positive selection towards the co-evolution of AS and functional domains. On the contrary, types II, III, and IV suggest that any kind of correlation, if existing, would be weak. The results obtained are shown in Table 3. We see that the first situation was never observed, in accordance with the very low probability of exact coincidence between AS and domain boundaries. However, the three remaining situations were found in both species with comparable ratios—including differences between observed and expected frequencies—indicating that the functional regulation of TFs by AS is reached by similar mechanisms in both species.

### 3.3. Expression of Orthologous TFs

**Figure 3:**
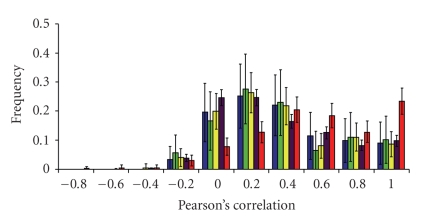
Comparison of the expression pattern of human and mouse ortholog TFs. The figure shows the distribution of Pearson's correlation values for the three following comparisons between human and mouse ortholog TFs: TFs having AS in both species (blue), TFs having AS in only one species (green) and TFs with no AS in any of the two species (yellow). In addition, it shows distribution for enzymes (purple) and replica's control (red). We calculated 95% confidence intervals for each bar on frequency histograms assuming the independence hypothesis [58].

All these results point to a coincidence between human and mouse in the regulation of TFs function by AS. Finally, we studied whether the presence/absence of AS may result in differences in TFs expression pattern. In our study we considered three different situations: (a) the TFs gene has AS in both species, (b) the TFs gene has AS in only one species; and (c) no AS was observed for the TFs genes in neither species. We compared TFs expression with that from enzymes, in order to have an external group. Because array data may show large fluctuations, we also used as a control experimental replicas for each TFs. When looking at the results (Figure 3) we can see that the replica's control has a high correlation in the expression distribution meaning that the different experiments were consistent. More interestingly, we observe that the distributions for the three TFs human-mouse comparisons are clearly different from that of the replica's control, whereas they are similar to that of enzymes. This points that, in general, human and mouse orthologous genes tend to have divergent tissue expression patterns and that TFs are no exception, even if the majority of their equivalent functional domains (either constitutive or alternative) are identical. This means that human and species do not use comparatively the same TFs in the same tissues. Finally, the trend does not depend on whether the TFs has AS, since the distributions for the three possible situations are essentially the same; thus, discarding the exclusive role of the specific isoforms in the apparition of the expression divergence. The uncoupling between AS and transcription regulation suggests they provide two independent levels of control of TFs products. 

Overall, our results indicate that rather than AS alone, a combination of AS and regulation at the transcription level may determine the nature and final amount of product for TFs [59, 60]. This suggests that in addition to changes at promoter regions, regulation of TFs activity might also play an important role in those processes that result in phenotypic differences between species. Importantly, this does not discard the role of other functional families or alternative phenomena, such as posttranslational modifications [61], in the apparition of interspecies differences. Additional experimental studies should be done in the future to test the validity of these hypotheses.

## Figures and Tables

**Table 1:** *Summary of AS data for human and mouse TFs*. The results for ALL GENES are those obtained analysing all entries in SwissProt. Each TFs dataset must be compared with the same species ALL GENES dataset.

	TFs		All genes	
	Human	Mouse	Human	Mouse

No. of genes	1077	737	12946	10031
% AS+	29.4	26.1	26.9	17.6
No. of isoforms/gene	3.0	2.9	2.8	2.7

**Table 2:** *Similarity among TFs*. The table shows the percentage of TFs isoforms which share an identity percentage. Control includes all the human non-TFs protein sequences.

	Control	Human	Mouse
No. of genes	10937	1077	737
No. of protein sequences	17026	1702	1093
90%	0.04	0.01	0.01
80%	0.07	0.02	0.02
70%	0.10	0.07	0.04
60%	0.16	0.13	0.08
50%	0.22	0.22	0.15
40%	0.31	0.34	0.23

**Table 3:** *AS effects on functional domains*. For each of the species are shown the observed and the expected (in parenthesis) frequencies.

	Human	Mouse
Type I	0.00 (0.00)	0.00 (0.00)
Type II	0.36 (0.20)	0.24 (0.14)
Type III	0.23 (0.42)	0.43 (0.47)
Type IV	0.41 (0.38)	0.33 (0.39)
